# Prevalence of HIV, syphilis, and condomless sex among MSM in Nepal; findings based on an integrated biological and behavioral surveillance survey using respondent-driven sampling

**DOI:** 10.1097/MD.0000000000046286

**Published:** 2025-11-28

**Authors:** Kiran Paudel, Kamal Gautam, Prashamsa Bhandari, Fiora Lena, Jeffrey A. Wickersham, Md. Safaet Hossain Sujan, Manisha Dhakal, Ran Xu, Tara Ballav Adhikari, Michael Copenhaver, Roman Shrestha

**Affiliations:** a Nepal Health Frontiers, Tokha-5, Kathmandu, Nepal; b Department of Allied Health Sciences, University of Connecticut, Storrs, CT; c Institute of Medicine, Tribhuvan University, Kathmandu, Nepal; d Yale School of Medicine, Department of Internal Medicine, Section of Infectious Diseases, New Haven, CT; e Blue Diamond Society, Kathmandu, Nepal; f Department of Public Health, Aarhus University, Aarhus, Denmark.

**Keywords:** condomless sex, HIV, men who have sex with men, Nepal, sexually transmitted infections, syphilis

## Abstract

In Nepal, men who have sex with men (MSM) encounter significant social stigma, which exacerbates their vulnerability to mental health issues. The interplay of these factors increases the likelihood of engaging in risky sexual behaviors, ultimately resulting in adverse health outcomes. This study assessed the prevalence of human immunodeficiency virus (HIV) and syphilis and factors associated with syphilis infection and condomless sex in the past 6 months among MSM in Nepal. We calculated HIV and syphilis prevalence and used bivariate and multivariate analyses to identify factors associated with syphilis infection and sexual risk. Among 250 participants, the weighted HIV prevalence was 2.6% (95% confidence interval [CI]: 0.6–4.6), and the syphilis prevalence was 13.1% (95% CI: 8.8–17.2). The practice of condomless sex was found to be in 71.4% (95% CI: 65.7–76.9) of the participants. Condomless sex in the past 6 months (respondent-driven sampling [RDS] weighted adjusted odds ratio [aOR]: 4.5; 95% CI: 1.1–18) and reported employment denial due to being MSM (RDS weighted aOR: 4.1; 95% CI: 1.5–10.8) were significantly associated with higher odds of syphilis infection. MSM who were single (RDS weighted aOR: 2.2; 95% CI: 1.1–4.4) and gay (RDS weighted aOR: 2.2; 95% CI 1.2–4.1) had a higher likelihood of having condomless sex in the past 6 months. These findings highlight the need for targeted interventions at multiple levels to address the specific needs of marginalized populations, such as MSM, to lower their risk of sexually transmitted infections and reduce unsafe sexual behaviors, notably unprotected sex.

## 1. Introduction

Sexually transmitted infections (STIs) remain a major public health issue globally, impacting millions each year. As of 2021, approximately 37.7 million people are affected by HIV, while 22.3 million individuals are impacted by syphilis.^[1]^ Among various key populations, such as men who have sex with men (MSM), the HIV epidemic is disproportionately prevalent, leading to a higher risk of such infections. Despite this, MSM are inadequately served and significantly impacted by the global response to HIV and Syphilis.^[2]^ Data from the United Nations Program on HIV/AIDS key population indicates that MSM have 28 times the risk of acquiring HIV compared to individuals of the same age group,^[3]^ likely because of their engaging in high-risk sexual behaviors such as condomless sex, having multiple sexual partners, and engaging in concurrent substance use.^[3,4]^ Nepal, too, identifies MSM as a key vulnerable population within its concentrated HIV epidemic and syphilis. According to estimates from 2020, the prevalence of HIV infection among MSM was significantly higher (3%) than the general population (<0.2%),^[5]^ and the prevalence of active syphilis was 4%.^[6]^

A major contributing factor to the high STI rates among MSM is the suboptimal use of condoms, which are 95% effective in preventing STIs, including HIV and syphilis transmission.^[7]^ Recent studies show that condom usage reduces the risk of HIV transmission during anal sex by 80% to 97%.^[8]^ However, data from the Centers for Disease and Control shows an increasing trend in condomless sex among MSM; condomless sex has increased by 24.5% among HIV-negative MSM and by 15.7% among HIV-positive MSM from 2012 to 2017.^[9]^ In Nepal, despite legislative protections and constitutional rights affirming the fundamental rights of sexual and gender minorities,^[10]^ MSM continue to confront stigma manifested in discrimination, exclusion, and ostracism from both their families and society at large.^[6,11,12]^ This social stigma has an economic impact that pushes some MSM into sex work, exacerbating their vulnerability to HIV and syphilis due to increased condomless sex with several sexual partners, some of whom may be unaware of their HIV/STI status.^[11,13]^ The stigma in Nepal against homosexuality also makes it difficult for MSM to access condoms, which ultimately increases their risk for HIV/STIs.^[13–15]^

There is limited research in Nepal on the prevalence of HIV, syphilis, and its risk factors. There is a lack of data regarding how this unique population, MSM in Nepal, are impacted by the prevalence of STIs and their risk factors (condomless sex). Addressing this issue is important as it can help develop preventive measures for HIV, syphilis, and their risk factors among Nepali MSM, reducing their vulnerability to these infections. Neglecting these issues can significantly impact the quality of life of sexual minorities, including MSMs, and increase their risk of STIs. We therefore conducted this study using RDS to examine the evolving trends in HIV and syphilis-related biological and risk factors among MSM in Nepal. In addition, the study aimed to assess the prevalence of HIV and syphilis and the implications of the risk factors experienced by MSM, including condomless sex. By employing the RDS approach, the research sought to capture a representative sample of the MSM population in the area and explore various factors associated with HIV and syphilis transmission.

## 2. Materials and methods

### 2.1. Study design and participants

Our study, named *Aayan* (translates as “gift of the god”), was a population-based bio-behavioral study conducted from October to December 2022 in the Kathmandu Valley in collaboration with the Blue Diamond Society. The Kathmandu Valley has 3 districts: Kathmandu, Bhaktapur, and Lalitpur. Participants who were at least 18 years old, understood Nepali or English, and were willing to undergo HIV and Syphilis screening were eligible for the study. The methodological details have been reported in an earlier publication from this study,^[16]^ and are briefly summarized here.

### 2.2. Recruitment and procedures

**Figure 1.**
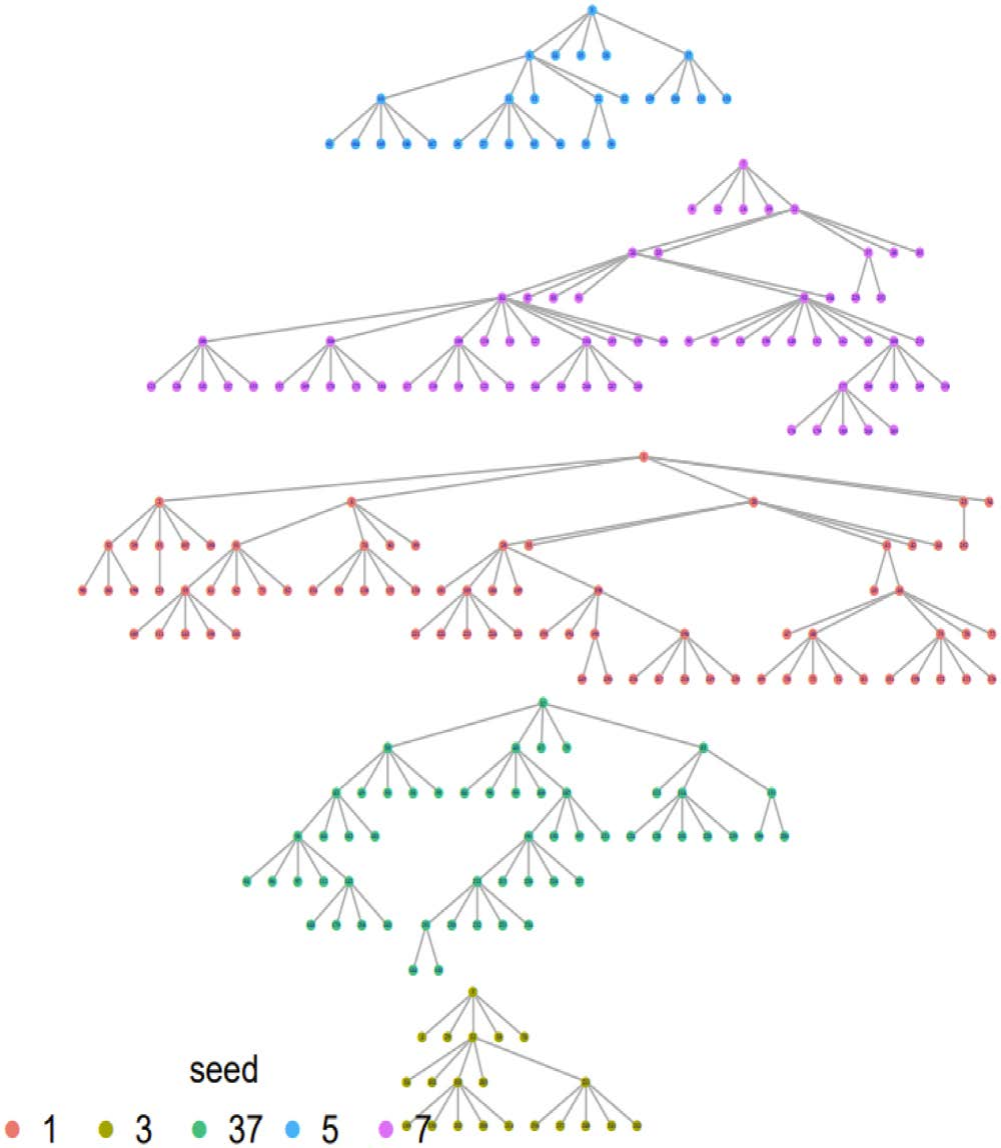
Respondent-driven sampling network diagram of the seeds and waves.

The recruitment of study participants in this research employed the respondent-driven sampling (RDS) technique, as shown in Figure 1. RDS is a network-based chain-referral sampling method used to recruit participants from populations that are hard to reach.^[17]^ The criminalization of homosexuality in many settings contributes to the significant stigma faced by MSM, especially in low- and middle-income countries (LMICs), driving them to conceal their sexual orientation. However, MSM often maintain strong social connections, creating a network of interrelationships that has been leveraged for public health research and surveillance.^[18,19]^ In this context, RDS has emerged as a valuable sampling strategy, designed explicitly for hidden populations like MSM. RDS operates by individuals recruiting their peers through reciprocal incentives.^[17]^ While RDS has predominantly been employed as a surveillance tool, its potential to identify infected individuals and facilitate their access to healthcare is hugely unrecognized. Very few studies validate the RDS method, particularly in developing countries.^[20]^

Recruitment began with 5 MSM “seeds” who were purposefully identified through community-based organizations familiar with local MSM networks. The selection of seeds ensured socio-demographic and geographic representation across the study sample.

After completing the interviewer-administered survey, each seed was provided with 5 uniquely coded recruitment coupons to invite peers from their social networks to participate. Subsequent participants who joined through these referrals were similarly given 5 coupons to continue the recruitment process, allowing the network to expand in successive waves. All participants provided informed consent prior to taking part in any study procedures, ensuring adherence to ethical standards throughout the research process.

Trained research assistants carried out in-person face-to-face interviews with the participants using the Qualtrics survey platform. All interviews were conducted in a private setting to ensure privacy, and each session lasted approximately 40 minutes, allowing for a comprehensive exploration of the research topics. Participants received compensation of 1000 Nepalese Rupees (approximately USD 8) for their participation. An additional incentive of 500 Nepalese Rupees (approximately USD 4) was provided for each eligible individual referred by a participant who subsequently enrolled in the study.

### 2.3. Ethics statement

The research protocol was reviewed and approved by the Institutional Review Board at the University of Connecticut (H22-0039) and by the Nepal Health Research Council (239/2022 P).

### 2.4. Study variables

The outcome variables considered for the study were the prevalence of HIV, syphilis, and condomless sex.

### 2.5. HIV and syphilis infection

Participants in the study received counseling and guidance both before and after undergoing testing for HIV and syphilis. Blood samples were collected to determine HIV and syphilis status. The testing method followed the Nepal national algorithm recommendation. Post-test counseling was provided on the same day to discuss the test results and offer support.

### 2.6. Condomless sex

Condomless sex is defined as sexual intercourse without using a condom or inconsistent condom use with either HIV-negative, positive, or unknown serostatus partners within the past 6 months. Participants’ responses were categorized into “No” (rarely, sometimes) or “Yes” (always).

### 2.7. Socio-demographic characteristics

The socio-demographic information collected for this study included age, birthplace by province, education, monthly income, religion, employment status, sexual orientation, relationship status, and living status. Age was divided into 2 groups: individuals younger than 25 years old and those 25 years old or older. Participants’ birthplaces were categorized into 2 groups: participants from provinces other than Bagmati were grouped as “outside Bagmati,” while participants from the Bagmati province were categorized separately as “Bagmati province.” Education levels were classified into 2 categories: up to SLC and higher secondary and above, where participants with higher secondary education or above were grouped. Monthly income was reported in Nepalese rupees and grouped into 2 categories: Rs. 20,000 (~ USD 150) or below and Rs. 20,000 and above. Sexual orientation was assessed as either gay or bisexual. Based on Nepal’s Health Management Information System categories, religious affiliation was categorized as Hindu, Buddhist, or other. Employment status was determined through a yes-or-no response indicating current employment. Relationship status was categorized as either single or with a partner. Living status was assessed based on 3 categories: living in one’s own home, living in a rented home, or other living arrangements.

### 2.8. HIV-related sexual risk behaviors

The assessment of HIV-related sexual risk behaviors included questions such as whether they had ever engaged in sex work, ever injected drugs, ever smoked, and used alcohol within the past 12 months. The perceived HIV risk was categorized into 2 groups: non-to-low risk and moderate to high risk. Participants were further asked to report the number of sexual partners they the past 6 months, which was grouped as multiple sex partners (yes/no).

### 2.9. Statistical analysis

The statistical software Stata.SE Corp version 17.0 was used for data analysis. Descriptive statistics were used to summarize the data, including frequency and percentages for categorical variables and mean and standard deviation for continuous variables.

To minimize potential biases associated with chain referral sampling, weighting adjustment were applied using RDS analysis Tool 7.1 (RDSAT, Cornell, NY). The RDSII estimator was used to account for the effect of differences in the participants’ social network sizes. Weights were based on the transition matrix for the dependent variable, current HIV status. Network size was determined based on participants’ response to 2 sequential questions: “How many men who have had oral or anal sex with men in the past 12 months do you know personally, who also know you and live in this city?” and “Among these men that you know personally, how many of them are 18 years and older?” The calculated weights were then exported from RDSAT and merged with the demographic and behavioral data in Stata for subsequent analyses.

Bivariate logistic regression models were used to estimate the unadjusted association between the outcome variables (HIV infection, syphilis infection, condomless sex), and the covariates selected based on prior empirical evidence and theoretical relevance from the published literature. RDS-weighted prevalence and bootstrapped confidence intervals were calculated for all variables explored in regression modeling. Multivariate logistic regression models were built to estimate the adjusted association between the current prevalence of HIV infection, syphilis infection, condomless sex, and the covariates. Bivariate and multivariate logistic regression models were also built with RDS weighting. *P*-values < .05 were used to indicate statistical significance.

## 3. Result

### 3.1. General characteristics of the study participants

Table 1 presents the RDS-weighted sociodemographic characteristics of the study participants. Of the 250 respondents, 54.1 (95% confidence interval [CI]; 47.8–60.3) were <25 years old, and 52.6 (95% CI; 46.4–58.8) studied at least higher secondary school. Among MSM, 50.9% (95% CI; 44.6–57.1) identified themselves as bisexual. Of the total respondents, 37.4% (95% CI; 31.3–43.4) were employed and more than half of the participants 51.3% (95% CI; 45.1–57.5) earned more than NRs. 20,000 and above. Most MSM rented a house rather than living in their own home; nearly two-thirds of the study participants were single.

**Table 1:** Crude and RDS-weighted prevalence of demographic characteristics among MSM in Nepal (N = 250).

Variables		N	Crude %	RDS weighted %, (95% CI)
Age groups (yr)				
	<25	127	50.8	54.1 (47.8–60.3)
	25 and above	123	49.2	45.9 (39.7–52.1)
Birthplace (by province)				
	Bagmati	148	59.2	60.7 (54.6–66.8)
	Outside Bagmati	102	40.8	39.3 (33.2–45.3)
Religion				
	Hindu	173	69.2	62.7 (56.7–68.7)
	Buddhist	54	21.6	26.0 (20.5–31.5)
	Others	23	9.2	11.3 (7.3–15.2)
Sexual orientation				
	Gay	158	63.2	49.1 (42.9–55.4)
	Bisexual	92	36.8	50.9 (44.6–57.1)
Educational status				
	Less than higher secondary	105	42.0	47.4 (41.2–53.6)
	Higher secondary and above	145	58.0	52.6 (46.4–58.8)
Employment status				
	Employee	106	42.4	37.4 (31.3–43.4)
	Unemployed	144	57.6	62.6 (56.6–68.7)
Income				
	Less than Nrs. 20,000	113	45.2	48.7 (42.5–54.9)
	Nrs. 20,000 and above	137	54.8	51.3 (45.1–57.5)
Relationship status				
	Single	161	64.4	67.2 (61.4–73.1)
	With partner	89	35.6	32.8 (26.9–38.6)
Living status				
	Living in own home	59	23.6	17.8 (13.1–22.6)
	Rented home and others	191	76.4	82.2 (77.4–86.9)

### 3.2. HIV, syphilis prevalence, and HIV-related sexual risk behavior

As shown in Table 2, the weighted prevalence of HIV among participants was 2.6% (95% CI; 0.6–4.6) and the syphilis prevalence was 13.1% (95% CI; 8.8–17.2). About one-fourth of the study participants had more than two sexual partners, and 71.4% (95% CI; 65.7–76.9) reported having condomless sex in the past 6 months. Only 26.4% (95% CI; 20.9–31.9) had ever injected drug, whereas nearly two third of the participants had ever smoked in their life and drank alcohol in the past 12 months.

**Table 2:** Crude and RDS weighted prevalence of HIV, syphilis, HIV-related sexual risk factors, drug risk behaviors, and mental health issues among MSM in Nepal (N = 250).

Variables		N	Crude %	RDS weighted %, (95% CI)
*Serology*				
HIV test				
	Positive	11	4.4	2.6 (0.6–4.6)
	Negative	238	95.6	97.4 (95.4–99.3)
Syphilis test				
	Positive	36	14.4	13.1 (8.8–17.2)
	Negative	214	85.6	86.9 (82.8–91.2)
*HIV-related sexual risk behaviors*				
	Ever engaged in sex work	55	22.0	13.7 (9.4–17.9)
	Multiple sex partners	101	40.4	26.9 (21.4–32.4)
	Condomless sex in the past 6 months	156	62.4	71.4 (65.7–76.9)
	Engaged in group sex work in past 6 months	36	14.4	8.7 (5.2–12.2)
	Perceived HIV risk			
	None to low	204	81.6	85.6 (81.3–90.0)
	Moderate to high	46	18.4	14.4 (10–18.7)
*Drug risk behaviors*				
	Ever injected drugs	70	28	26.4 (20.9–31.9)
	Drank alcohol in past 12 months	179	71.6	63.8 (57.8–69.7)
	Ever smoke	182	72.8	67.6 (61.8–73.5)
*Mental health issue*				
	Depressive symptoms	49	19.6	11.7 (7.7–15.7)

### 3.3. Individual and socio-structural factors associated with syphilis

Condomless sex in the past 6 months (RDS weighted aOR: 4.5; 95% CI: 1.1–18) and reporting employment denial due to being MSM (RDS weighted aOR: 4.1; 95% CI: 1.5–10.8) were significantly associated with higher odds of syphilis infection as shown in Table 3.

**Table 3:** RDS weighted associations between independent variables and syphilis among MSM (N = 250).

Variables		RDS adjusted odds ratio	95% CI	*P* value
Educational status				
	Higher secondary school and above	2.2	0.9–5.4	.08
	Less than higher secondary school	Ref		
Living Status				
	Rented home and other	0.8	0.3–2.2	.6
	Living in own home	Ref		
Condomless sex in the past 6 months				
	Yes	4.5	1.1–18.4	**.04**
	No	Ref		
Ever injected drug				
	Yes	0.3	0.1–1.1	.05
	No	Ref		
Multiple sex partners				
	Yes	Ref		
	No	0.5	0.2–1.5	.2
Belief that MSM are not accepted in Nepali Society				
	Yes	1.8	0.7–4.7	.2
	No	Ref		
Ever denied employment for being MSM				
	Yes	4.1	1.5–10.8	**.006**
	No	Ref		
Sexually abused for being MSM				
	Yes	1.7	0.3–10.5	.5
	No	Ref		

### 3.4. Individual and socio structural factors associated with condomless sex

The multiple logistic regression model revealed that MSM who identify themselves as single (RDS weighted adjusted odds ratio [aOR]: 2.2; 95% CI: 1.1–4.4) and Gay (RDS weighted aOR: 2.2; 95% CI 1.2–4.1) had a higher likelihood of having condomless sex as shown in Table 4. Participants with “no” to “low” perceived risk of getting infected with HIV (RDS weighted aOR: 3.2; 95% CI 1.2–8.6) had higher odds of having condomless sex. Additionally, MSM who had multiple sexual partners (RDS weighted aOR: 2.2; 95% CI 1.1–4.2) had a higher likelihood of engaging in condomless sex.

**Table 4:** RDS weighted associations between independent variables and condomless sex (N = 250).

Variables		RDS adjusted odds ratio	95% CI	*P* value
Sexual orientation				
	Gay	2.2	1.2–4.1	.01
	Bisexual	Ref		
Relationship status				
	Single	2.2	1.1–4.4	.02
	With partner	Ref		
Perceived HIV risk				
	None-low	3.2	1.2–8.6	.02
	Moderate-high	Ref		
Not accepted in society				
	Yes	0.6	0.3–1.1	.09
	No	Ref		
Drank alcohol in the last 12 months				
	Yes	2.2	0.8–3.2	.17
	No	Ref		
Multiple sexual partners				
	Yes	2.2	1.1–4.2	.02
	No	Ref		

## 4. Discussion

This study assessed the prevalence of HIV and syphilis and its risk factors (condomless sex) among MSM in Nepal. We found that the RDS-weighted prevalence of HIV was 2.6%, the prevalence of syphilis was 13.1% and condomless sex in the past 6 months was 71.4%. Notably, the study found a markedly higher prevalence of syphilis compared to previous research conducted among Nepali MSM in the Terai region.^[6]^ Although the Terai highway districts in Nepal are recognized as “hot-spots” for HIV due to the open border with India, which facilitates sex and drug trafficking, however, it is important to acknowledge that MSM in Kathmandu Valley also encounter a similar environment to that of the border regions.^[21]^

The findings of this study revealed that a significant proportion of MSM (71.4%) reported to have engaged in condomless sex. Not surprisingly, MSM who engaged in condomless sex had a higher likelihood of syphilis which is consistent with findings from prior studies.^[22–24]^ Prior research has consistently shown high prevalence of condomless anal sex among MSM^[6,7]^ that puts them at increased risk of STIs, including HIV.^[22–24]^ Furthermore, factors such as substance use and participation in chemsex, both of which are prevalent in MSM and are known to impair judgment, further increase the likelihood of engaging in condomless sex and having multiple sexual partners.^[25–28]^ This suggests that the relatively high rate of condomless anal sex, which is frequent among MSMs, is a significant risk for the continued spread of STIs, including HIV.

Our findings indicate that MSM who face employment discrimination had higher likelihood of contracting syphilis. Several studies have consistently reported high rates of unemployment and employment discrimination among sexual and gender minority individuals,^[11,29]^ leading to greater levels of economic inequality. This lack of financial stability can be associated with increased stress and a sense of instability, potentially leading some individuals to engage in risk-taking behaviors, including risky sexual practices. Additionally, unemployment may push individuals towards alternative economic strategies, such as involvement in sex work,^[30]^ further exacerbating their vulnerability to contracting STIs such as syphilis.

Furthermore, our findings showed that single MSM were more likely to engage in condomless sex compared to those in committed relationships, as indicated by previous studies.^[7,31,32]^ They may be less inclined to discuss sexual health and condom use with casual partners, often viewing condoms as a hindrance to spontaneous sexual encounters.^[7,33]^ Conversely, MSM in relationships are typically more informed about each other’s HIV and other STI statuses, enabling safer sexual practices. Given these dynamics, it is imperative to educate both single and partnered MSM not only about the consistent use of condoms but also about the effectiveness of other preventive measures such as PrEP.^[34]^ Additionally, it is crucial to communicate the Undetectable = Untransmittable (U = U) principle, which assures that HIV-positive individuals with an undetectable viral load cannot sexually transmit the virus.^[35,36]^ However, it is vital to emphasize that PrEP and an undetectable viral load do not protect against other STIs. Therefore, condom use remains the most effective method to prevent STIs such as chlamydia, gonorrhea, and syphilis, allowing both single and partnered MSM to make more informed decisions about safer sex practices.

Our study found that MSM who perceived themselves to be at low risk for HIV infection were more likely to engage in condomless sex. This pattern is consistent with the established health behavior theories, such as the health belief model and protection motivation theory, which posit that risk perception is a critical precursor in adopting health-promotive and protective behaviors.^[37,38]^ However, perceived risk does not always reflect the actual risk; studies indicate that individuals may underestimate their HIV risk due to factors like knowledge gaps and optimism bias, leading to less frequent testing and lower engagement in prevention behaviors^[39–41]^ Thus, interventions should aim not only to raise HIV risk awareness and promote condom use among MSM but also to reconcile these perceptions with actual risks through targeted education and personalized risk assessments. Interestingly, participants who have multiple sexual partners had a higher likelihood of participating in condomless sex. Previous studies from MSM in China support our claim that MSM in multiple sexual relationships have higher odds of partaking in condomless sex.^[22]^ One possible reason for condomless sex with multiple sexual partners could be because of educational discrepancies. These education discrepancies are due to the homophobic nature of society, as school systems do not teach about homosexual sex safety.^[22]^ This stems from the fact that homosexuality is forbidden, therefore there is limited knowledge of how to engage in homosexual intercourse. There is a lack of STI knowledge throughout the MSM community, as anal intercourse is forbidden and not taught in schools. Without an educational initiative to understand anal intercourse and its risks, many MSM are unaware of its implications. The social perception that using condoms is a symbol of distrust and the topic of STIs can be considered an embarrassing topic in Nepal as well.^[22]^ The more sexual partners an individual has, the more likely they are to become embarrassed to bring up the topic of STI prevention with each sexual partner, as their relationship may purely be sexual. Leveraging mHealth interventions, including community-designed mobile applications that offer sexual health education, risk assessments, STI screening reminders, and anonymous healthcare consultations, presents a promising strategy to enhance sexual health awareness, promote safer practices, and address stigma-related barriers among tech-savvy younger MSM in Nepal.

There are several limitations of the study that need to be addressed in further similar studies. Firstly, due to the cross-sectional nature of the study design, we cannot infer causality. Secondly, our study was conducted in Kathmandu Valley, Nepal, and the sample did not represent populations from other areas in Nepal. Thirdly, the sample size was modest. Therefore, some of the nonsignificant associations observed may have occurred due to inadequate power. Thus, longitudinal studies with larger samples are required to confirm and verify our findings. Despite its limitations, this study used respondent-driven sampling methods to increase the representatives of our sample. Also, during the analysis, RDS weighted value and network analysis were performed, which helped in actual predictions of value. We measured HIV and Syphilis from blood samples in the lab; therefore, the main objectives of this study to measure prevalence of HIV and syphilis were neither overestimated nor underestimated.

## 5. Conclusion

In Nepal, MSM face discrimination, resulting in both individual and system factors that impact their susceptibility to STIs, including HIV and syphilis. These findings highlight the need for targeted interventions at multiple levels to address the specific needs of marginalized populations such as MSM to lower their risk of STIs and reduce unsafe sexual behaviors, notably unprotected sex.

## Author contributions

**Conceptualization:** Kiran Paudel, Kamal Gautam, Jeffrey A. Wickersham, Roman Shrestha.

**Data curation:** Kiran Paudel, Kamal Gautam, Prashamsa Bhandari, Ran Xu, Roman Shrestha.

**Formal analysis:** Kiran Paudel, Kamal Gautam, Roman Shrestha.

**Methodology:** Kiran Paudel, Kamal Gautam, Prashamsa Bhandari, Fiora Lena, Jeffrey A Wickersham, Manisha Dhakal, Tara Ballav Adhikari, Roman Shrestha.

**Project administration:** Kiran Paudel, Kamal Gautam, Jeffrey A. Wickersham, Manisha Dhakal, Roman Shrestha.

**Resources:** Kiran Paudel, Kamal Gautam, Jeffrey A. Wickersham, Manisha Dhakal, Ran Xu, Michael Copenhaver, Roman Shrestha.

**Software:** Kiran Paudel, Ran Xu.

**Visualization:** Kiran Paudel.

**Writing – original draft:** Kiran Paudel, Kamal Gautam, Prashamsa Bhandari, Fiora Lena, Jeffrey A. Wickersham, Md. Safaet Hossain Sujan, Ran Xu, Tara Ballav Adhikari, Michael Copenhaver, Roman Shrestha.

**Writing – review & editing:** Kiran Paudel, Kamal Gautam, Prashamsa Bhandari, Fiora Lena, Jeffrey A. Wickersham, Md. Safaet Hossain Sujan, Manisha Dhakal, Ran Xu, Tara Ballav Adhikari, Michael Copenhaver, Roman Shrestha.

**Supervision:** Kamal Gautam, Jeffrey A. Wickersham, Manisha Dhakal, Ran Xu, Tara Ballav Adhikari, Michael Copenhaver, Roman Shrestha.

**Validation:** Ran Xu, Tara Ballav Adhikari, Michael Copenhaver.

**Investigation:** Roman Shrestha.
